# Expression of the Biofilm-Associated Genes in Methicillin-Resistant *Staphylococcus aureus* in Biofilm and Planktonic Conditions

**DOI:** 10.3390/ijms19113487

**Published:** 2018-11-06

**Authors:** Barbara Kot, Hubert Sytykiewicz, Iwona Sprawka

**Affiliations:** 1 Department of Microbiology, Faculty of Natural Sciences, Siedlce University of Natural Sciences and Humanities, 14 Bolesława Prusa Str., 08-110 Siedlce, Poland; 2 Department of Biochemistry and Molecular Biology, Faculty of Natural Sciences, Siedlce University of Natural Sciences and Humanities, 14 Bolesława Prusa Str., 08-110 Siedlce, Poland; hubert.sytykiewicz@uph.edu.pl (H.S.); iwona.sprawka@uph.edu.pl (I.S.)

**Keywords:** MRSA, biofilm, expression of genes, quantitative real-time PCR

## Abstract

The role of genes that are essential for development of *Staphylococcus aureus* biofilm during infection is not fully known. mRNA from two methicillin-resistant *S. aureus* strains that formed weak and strong biofilm on polystyrene plates were isolated at five time points from cells grown in biofilm and planktonic culture. Quantitative real-time PCR analysis showed that the expression levels of investigated genes under biofilm conditions were significantly higher than under planktonic conditions. The expression levels of the gene encoding elastin binding protein (*ebps*) and laminin binding protein (*eno*) were significantly increased in biofilm at 3 h, both in strongly and weakly adhering strain. The peak expression of *fib* gene encoding fibrinogen binding protein was found at 6 and 8 h in the case of strongly and weakly adhering strain, respectively. The expression of *icaA* and *icaD* genes in both strains was significantly higher under biofilm conditions when comparing to planktonic cells during 12 h. The expression level of the genes encoding binding proteins and the glucosamine polymer polysaccharide intercellular adhesin (PIA) slowly decreased after 24 h. Finally, we found that the expression levels of genes encoding binding factors in weakly adhering strain were significantly lower than in strongly adhering strain.

## 1. Introduction

Methicillin-resistant *Staphylococcus aureus* (MRSA) are responsible for many diseases from skin infections to invasive infections, such as pneumonia, infections of soft tissues, bones, heart valves, and even fatal septicemia in human [1]. The number of infections caused by MRSA isolates increased during the recent years. MRSA are recognized as resistant to β-lactam antibiotics but often the MRSA strains are multiresistant because they show resistance to different classes of antimicrobials. *S. aureus* is one of the most common causes of bacteremia. Currently, this microorganism is responsible for 20–40% mortality at 30 days, despite appropriate treatment [2]. Chronic infections, often having a biofilm nature, are serious medical threat. Biofilm consists of multiple layers of bacteria enclosed in a self-produced exopolysaccharide glycocalyx protecting the bacteria from host defenses and being a barrier for some antibiotics. Bacterial cells within the biofilm display phenotypic diversity and variable levels of metabolic activity due to the differential diffusion of nutrients and water through the biofilm structure [3]. Bacteria within biofilm adopt a phenotype that confers resistance to large variety of antimicrobial agents [4]. The formation of biofilm is a complex process that can be subdivided into phases of attachment, accumulation, maturation, and dispersal. The first step of staphylococcal infection is the attachment of bacterial cells to surfaces of various materials, including host tissues and medical devices and the accumulation of bacterial cells. Attachment is mediated by different types of adhesins [5]. Many of these adhesins have the ability to recognize large glycoproteins found in the host plasma and extracellular matrix. The functional diversity of these adhesins contributes to the ability of *S. aureus* to adapt to various microenvironments as connective tissue, bone, the bloodstream, and vascular tissue. The group of surface-exposed proteins expressed by *S. aureus* are MSCRAMMs (microbial surface components recognizing adhesive matrix molecules) that are responsible for the initial attachment to native tissues and biomaterials [6]. These MSCRAMMs can bind to one or more host extracellular matrix factors including laminin, elastin, and fibrinogen [7]. Whereas, main adhesin responsible for accumulation phase is the polysaccharide intercellular adhesin (PIA) that is the primary determinant promoting adhesive interactions between bacterial cells and is composed of β-1,6-linked N-acetylglucosamine residues and an anionic fraction with a lower content of non-*N*-acetylated D-glucosaminyl residues [8,9]. PIA is encoded by *ica* operon the expression of which is regulated by environmental factors such as anaerobic growth conditions inducing PIA production [10]. The products of the *ica* operon comprising *icaADBC* genes were demonstrated to be necessary for biofilm formation [11]. IcaA alone has low *N*-acetylglucosaminyl-transferase activity. Whereas, co-expression of *icaA* and *icaD* increases about 20-fold *N*-acetylglucosaminyl transferase activity and slime production [12]. Because the role of genes that are essential for the development of biofilms during infection is interesting and not fully understood, we investigated the transcriptional profiles of specific staphylococcal genes (*eno*, *ebps*, *fib*) encoding MSCRAMMs and genes from *ica* operon (*icaA* and *icaD*) in five time points during biofilm production on polystyrene plates and under planktonic conditions for two strains differing in the ability of biofilm formation. The *eno* gene encodes α-enolase, which is expressed at the surface of the bacterial wall. It was demonstrated that α-enolase is able to bind laminin and also functions as a plasminogen receptor. α-enolase can mediate the binding of *S. auresus* to laminin, allowing bacterial cells adherence to the extracellular matrix and initiating tissue colonization in different sites in the host. Laminin is a major component of the basal membrane of the vasculature. Adherence to laminin may contribute to tissue invasion and dissemination of staphylococcal cells by blood. Besides, α-enolase by plasminogen activation can cause laminin degradation in restricted areas [13]. The elastin binding protein of *S. aureus* (EbpS) is a cell-surface-associated 25 kDa protein encoded by the *ebps* gene. Binding *S. aureus* by EbpS to the N-terminal 30 kDa region of elastin which is a major component of the elastic fiber extracellular matrix, promote colonization of mammalian tissues by *S. aureus* [14]. *S. aureus* express three receptors for fibrinogen. One of them is fibrinogen binding protein Fib, which is encoded by *fib* gene [15]. Fibrinogen is a glycoprotein that is present in the blood that mediates platelet adherence and aggregation at injury site. Fibrinogen is one of the main proteins deposited on implanted biomaterials. The ability of *S. aureus* to adhere to fibrinogen is an important factor in promoting wound infection, foreign body infection, and endocarditis [16]. Understanding of biofilm development requires the knowledge of genes participating in this process and timing of their expression. Besides, knowledge about gene expression of *S. aureus* during biofilm formation is needed to understand the high persistence and resistance of biofilm cells, which can be important in search of effective treatments of infections associated with biofilms.

## 2. Results

### 2.1. PCR Assay

**Figure 1:**
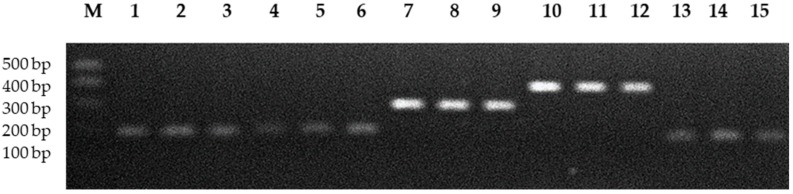
Electrophoresis in 1.5% agarose gel PCR products obtained by using specific primers for *icaA*, *icaD*, *eno*, *fib* and *ebps* genes. Lines M: molecular weight marker (500, 400, 300, 200, 100 bp; GenoPlast Biochemicals, Poland); Lines 1–3: products (188 bp) specific for *icaA* gene; Lines 4–6: products (198 bp) specific for *icaD* gene; Lines 7–9: products (302 bp) specific for *eno* gene; Lines 10–12: products (404 bp) specific for *fib* gene; and, Lines 13–15: products (186 bp) specific for *ebps* gene.

Based on the primers used in the experiment three MSCRAMM genes (*eno*, *ebps* and *fib*) and two genes from *ica* operon (*icaA*, *icaD*) were detected in all *S. aureus* strains. The specific PCR products for these genes are shown in Figure 1.

The presence of the *mecA* gene responsible for resistance of *S. aureus* to methicillin was also detected in all strains (Table 1).

### 2.2. Biofilm Formation

**Figure 2:**
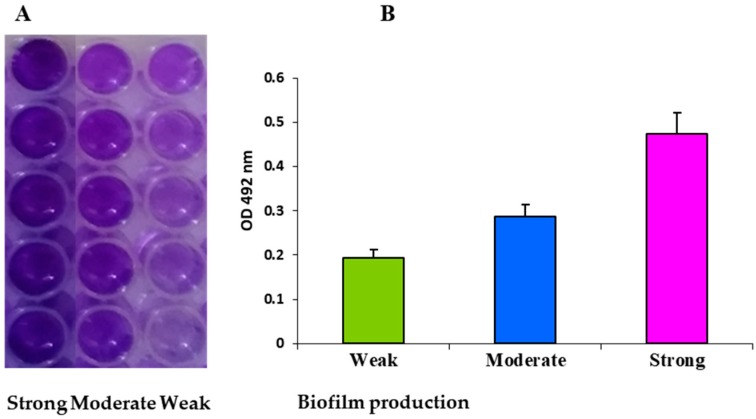
Biofilm formation on polystyrene 96-well plates by methicillin-resistant *Staphylococcus aureus* (MRSA) strains. The weak, moderate and strong biofilm on polystyrene microtiter plate surface stained with 1% crystal violet after 48 h of incubation MRSA strains (**A**). The quantitative analysis of biofilm production by measuring the optical density of stained biofilms at 492 nm (**B**). Whiskers indicate standard deviations.

The investigated MRSA strains showed the different degree of adherence to polystyrene microtiter plates (Table 1, Figure 2A). The strain that was isolated from wound formed strong biofilm on polystyrene with OD_492_ values of 0.474 ± 0.06 after 48 h of incubation. The strain from the respiratory tract adhered to polystyrene in moderate degree (OD_492_ = 0.286 ± 0.05), while strain isolated from anus in weak degree (OD_492_ = 0.193 ± 0.02) (Figure 2B).

### 2.3. Expression Levels of Genes Associated with Biofilm Formation Quantified by qRT-PCR

**Figure 3:**
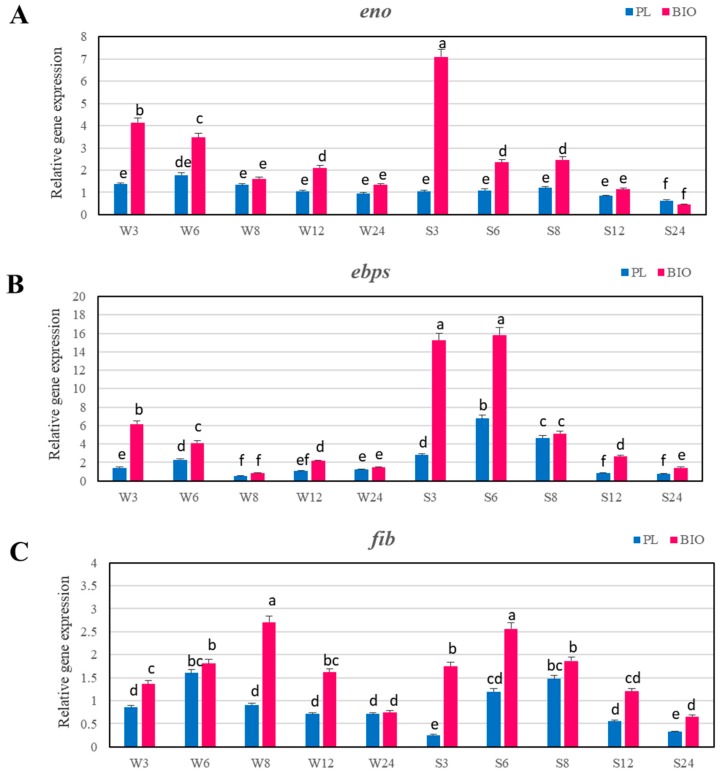
Transcriptional profiling of *eno* (**A**), *ebps* (**B**), and *fib* (**C**) genes in the examined MRSA strains in planktonic growth (PL) and biofilm conditions (BIO). Data were normalized to *rpoB* (reference) gene. Results are presented as n-fold changes ± standard deviation (SD) in relation to the calibrator (planktonic cells at 6 h of growth period of MRSA strain no. 156, which was the moderate biofilm producer). Different letters (a, b, c, d, e, f, de, ef, cd, bc) denote significant differences in target gene expression among investigated MRSA samples (Tukey’s test; *p* < 0.05). W—weak producer of biofilm; S—strong producer of biofilm; 3, 6, 8, 12, 24—time of bacterial growth (h).

Expression levels of five genes that are involved in biofilm formation were investigated for two MRSA strains selected based on a different degree of adhesion to polystyrene surface. MRSA strain isolated from wound produced strong biofilm, while the strain from anus was a weak biofilm producer. As the calibrator sample planktonic cells of MRSA strain from respiratory tract showing moderate biofilm formation were used. The results are presented as n-fold changes in the target gene expression, in relation to the calibrator. The expression patterns for these genes were followed after 3, 6, 8, 12, and 24 h of growth, and they were compared between biofilm and planktonic growth conditions. In the group of genes encoding MSCRAMMs that are responsible for the initial attachment of *S. aureus*, the expression level of *ebps* (elastin binding protein), and *fib* (fibrinogen binding protein) in strongly adhering strain was highest under biofilm conditions during first 6 h, while the expression level of *eno* gene (laminin binding protein) was highest after 3 h (Figure 3A–C).

**Figure 4:**
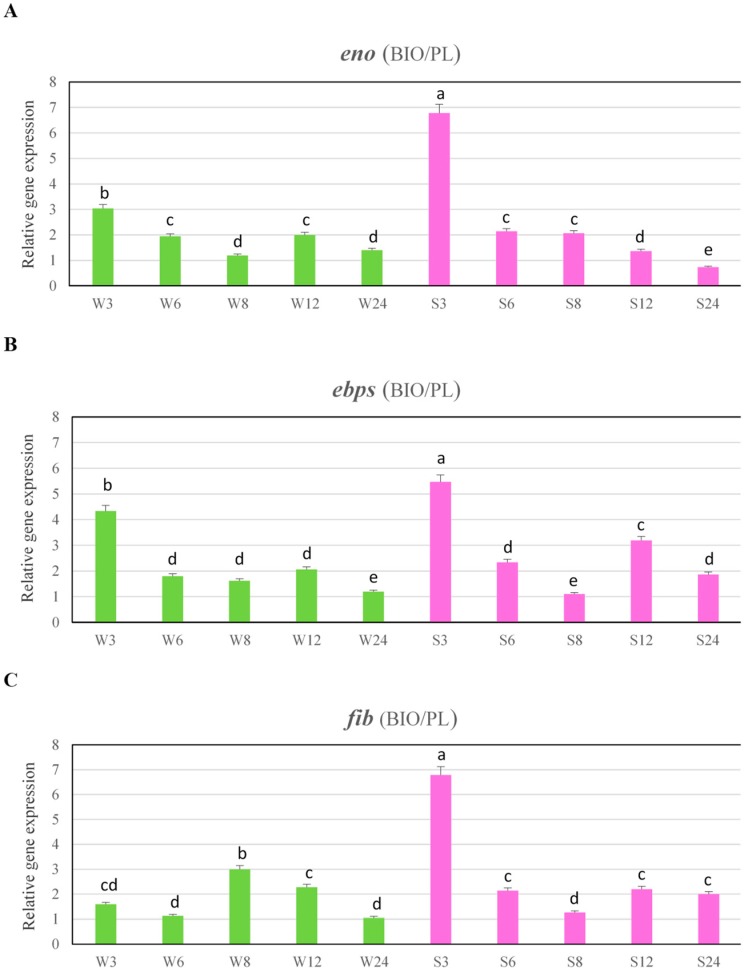
Abundance of *eno* (**A**), *epbs* (**B**), and *fib* (**C**) transcripts under biofilm conditions (BIO) versus planktonic cells (PL). Data were normalized to *rpoB* (reference) gene. Results are presented as n-fold changes ± standard deviation (SD) in relation to the calibrator (planktonic cells at 6 h of growth period of MRSA strain no. 156, which was the moderate biofilm producer). Different letters (a, b, c, d, e, cd) denote significant differences in target gene expression among investigated MRSA samples (Tukey’s test; *p* < 0.05). W—weak producer of biofilm; S—strong producer of biofilm; 3, 6, 8, 12, 24—time of bacterial growth (h).

The expression levels of *ebps*, *fib* and *eno* in these time intervals were significantly higher in biofilm cells than in planktonic cells. In the next time point, the expression levels of these genes in strongly adhering strain decreased, but at 6 and 8 h in the case of *eno* and at 12 and 24 h in the case of *ebps* were significantly higher under biofilm conditions than under planktonic conditions. In weakly adhering strain the expression levels of genes encoding binding factors were significantly lower than in strongly adhering strain, but in almost of all time intervals, they were significantly higher under biofilm conditions than in planktonic cells. In this strain the expression levels of *eno* and *ebps* genes under biofilm conditions were the highest during first 6 h (Figure 3A,B) and in the case of *fib* gene, the expression growing slower and peaked at 8 h (Figure 3C). The expression levels of genes encoding binding factors in case of strongly adhering strain after 3 h were about 6- (*ebps*) to 7-fold (*eno* and *fib*) higher under biofilm conditions than under planktonic conditions. While, in weakly adhering strain, the expression levels of *eno* and *ebps* were only 3- (*eno*) and above 4 (*ebps*) times higher under biofilm conditions than under planktonic conditions. The highest expression level of *fib* gene in weakly adhering strain at 8 h was three-fold higher under biofilm conditions than in planktonic cells (Figure 4A–C).

**Figure 5:**
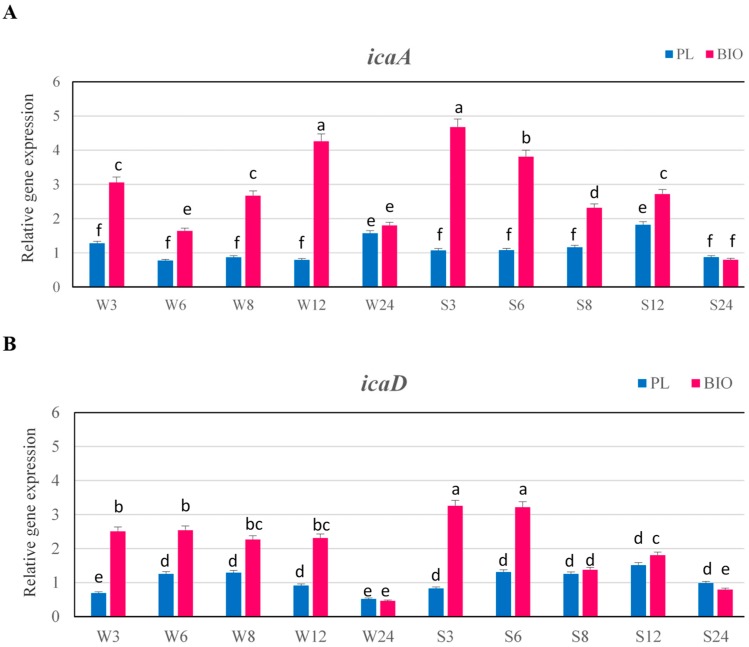
Transcriptional profiling of *icaA* (A) and *icaD* (B) genes in the examined MRSA strains in planktonic growth (PL) and biofilm conditions (BIO). Data were normalized to *rpoB* (reference) gene. Results are presented as n-fold changes ± standard deviation (SD) in relation to the calibrator (planktonic cells at 6 h of growth period of MRSA strain no. 156, which was the moderate biofilm producer). Different letters (a, b, c, d, e, f, bc) denote significant differences in target gene expression among investigated MRSA samples (Tukey’s test; *p* < 0.05). W—weak producer of biofilm; S—strong producer of biofilm; 3, 6, 8, 12, 24—time of bacterial growth (h).

The expression levels of genes from *ica* operon (*icaA* and *icaD*) that are involved in biosynthesis of glucosamine polymer PIA were also evaluated. In the strongly adhering strain, the expression levels of *icaA* and *icaD* under biofilm conditions were highest after 3 and 6 h, and significantly higher than in planktonic culture (Figure 5A,B).

**Figure 6:**
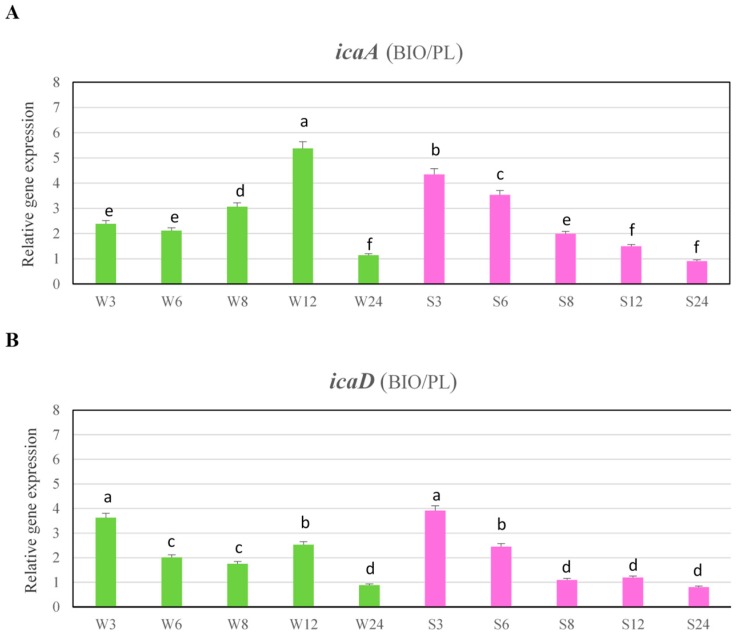
Abundance of *icaA* (**A**) and *icaD* (**B**) transcripts under biofilm conditions (BIO) versus planktonic cells (PL). Data were normalized to *rpoB* (reference) gene. Results are presented as n-fold changes ± standard deviation (SD) in relation to the calibrator (planktonic cells at 6 h of growth period of MRSA strain no. 156, which was the moderate biofilm producer). Different letters (a, b, c, d, e, f) denote significant differences in target gene expression among investigated MRSA samples (Tukey’s test; *p* < 0.05). W—weak producer of biofilm; S—strong producer of biofilm; 3, 6, 8, 12, 24 – time of bacterial growth (h).

The expression levels of these genes at 3 h were four-fold higher in biofilm cells than in planktonic growth (Figure 6A,B).

In the strongly adhering strain, the expression levels of *icaA* and *icaD* genes declined after 8 and 12 h, but they were higher under biofilm conditions than under planktonic conditions.

In the weakly adhering strain the expression level of *icaA* gene in biofilm was always significantly higher than under planktonic conditions and the highest expression level in biofilm was detected at 12 h and it was 5.4-fold higher than in planktonic cells. While, the expression level of *icaD* gene in weakly adhering strain did not significantly change over initial 12 h of biofilm growth, but it was significantly higher than under planktonic conditions. After 24 h, the expression level of *icaA* and *icaD* genes in weakly adhering strain was significantly lower and it did not significantly differ from expression level under planktonic conditions.

Importantly, factorial ANOVA results indicate that tested variables (strains, cell forms, growth period), as well as their interactions significantly affected expression levels of five target genes (*icaA*, *icaD*, *eno*, *ebps*, *fib*) of MRSA strains (Table S1).

## 3. Discussion

*S. aureus* is an important species, including potentially pathogenic strains. Infections that are caused by *S. aureus* are difficult to treat because of the ability of biofilm formation on biological surfaces, which allows *S. aureus* strains to efficiently colonize the host organism. Infections caused by *S. aureus* which are associated with formation of biofilm include endocarditis, osteomyelitis, septic arthritis and infections associated with implanted medical devices [11]. The formation of biofilm facilitates bacterial colonization of host organism and also provides resistance to antibiotics and the host immune system. Knowledge concerning expression of genes responsible for adhesion and biofilm formation is critical for understanding how *S. aureus* causes infectious diseases and it can be helpful in the prevention and treatment of these diseases. To our knowledge, the timing and the relative quantification of the gene expression that is involved in biofilm formation by MRSA strains was investigated only by Atshan et al. [17]. The DNA microarray analysis and RT-PCR results of *S. aureus* biofilm gene expression were also reported by other authors [4,18,19] but the knowledge about the participation of particular genes in the process of biofilm formation remains largely unknown.

In our research, we investigated in vitro expression of representative genes of binding factors and of genes involved in the biofilm formation. Among the genes of binding factors we investigated the *eno*, *ebps* and *fib* genes, encoding the laminin, elastin, and fibrinogen binding protein, respectively. We demonstrated a quantitative PCR analysis of their expression in two MRSA strains differing in the degree of biofilm formation, which were grown in biofilm and under planktonic conditions for 3, 6, 8, 12, and 24 h. Among investigated adhesin genes, the expression level of *ebps* gene, encoding elastin binding protein, was highest under biofilm conditions during first 6 h in relation to the calibrator, both in the case of weakly and strongly adhering strain. The expression level of *ebps* after 3 h was about 5.5-fold higher under biofilm conditions than under planktonic conditions in strongly adhering strain and 4.3-fold higher in the weakly adhering strain. The results presented in our study are contradictory to the results obtained by Atshan et al. [17] who showed that the expression level of *ebps* gene was significantly enhanced more than 6-fold at 24 and 48 h compared to 12 h. In our study, the expression level of this gene decreased in the next time points but after 24 h in case of strongly adhering strain under biofilm conditions was also significantly higher than under planktonic conditions but only about two-fold. Elastin is an insoluble polymeric fiber of the extracellular matrix responsible for reversible elasticity of tissues and organs. That’s why elastin is abundant in lung, skin, and major blood vessels but elastin is also widely expressed at lower levels in most mammalian tissues. The *ebps* gene product is an integral membrane protein of *S. aureus* that binds to elastin and this interaction may promote bacterial colonization and facilitate pathogenesis [14]. Similar results as expression level of *ebps* gene we also obtained for two remaining genes encoding MSCRAMMs that are responsible for the initial attachment of *S. aureus*. The expression of surface receptors encoded by *eno* gene that specifically recognize the protein laminin of basement membranes (BM) has been correlated with the capacity of bacterial cells to bind and cross the barrier of BM [13]. The fibrinogen binding protein encoded by *fib* gene is an important adherence factor responsible for the ability of *S. aureus* to adhere to fibrinogen adsorbed on catheters and endothelial cells [16]. In our research, the expression level of *eno* gene after 3 h was above seven-fold higher in biofilm than under planktonic conditions in the case of strongly adhering strain and over three-fold higher in in the case of weakly adhering strain. The expression level of this gene after 12 h was similar to the results that were obtained by Atshan et al. [17]. In this study, the peak expression of the *fib* gene was observed after 6 h in the case of strongly adhering strain and after 8 h in the case of weakly adhering strain; the expression levels were about seven-fold and three-fold higher in biofilm than under planktonic conditions at this time, respectively. In the next time points, the expression level of these genes decreased, although after 12 h in biofilm it was significantly higher than under planktonic conditions. These results are contrary to those that were obtained by Atshan et al. [17], who showed that the expression level of *fib* gene did not differ from control or slightly increased after 24 h in case of some investigated strains.

In our opinion, the significantly higher transcript levels of *ebps*, *eno,* and *fib* in the first hours of growth under biofilm conditions compared to growth under planktonic conditions suggest that these genes are important for the first phase of biofilm growth, in which bacterial cells interact with host extracellular ligands.

The ability to form biofilm requires also accumulation of bacterial cells to form multilayered cell clusters which are embedded in a slime substance. The expression of *ica* genes leading to the synthesis of the glucosamine polymer PIA [8] is important for the adhesion and formation of staphylococcal biofilms. It was interesting to determine whether the *icaA* and *icaD* genes involved in biosynthesis of PIA are expressed at higher levels in the biofilm of MRSA strains and in which phase of biofilm growth. The results of our research showed that the expression of *icaA* and *icaD* genes in both strains was significantly higher under biofilm conditions than under planktonic conditions during 12 h, although, in case of strongly adhering strain, the peak of expression was detected at 3 and 6 h, which is in concordance with the results that were obtained by Resch et al. [19] who found that after 6 and 8 h of growth in biofilm cells the *icaA* and *icaD* were expressed at highest levels, higher than in planktonic cells. In our study, the expression level declined at later growth stages, which is also similar to the results that were obtained by Resch et al. [19]. In this study, as in the case of genes encoding MSCRAMMs, the *ica* genes are up-regulated at the beginning of biofilm formation. This indicates that products of these genes are associated with the initial colonization phase during biofilm formation by *S. aureus*, rather than maturation and its persistence. At the later colonization stage, when the cells are already attached to the surface and biofilm formation begins and growing of cells is retarded due to nutrient depletion, increase of expression levels of these genes is probably no more necessary. Dobinsky et al. [20] showed that *ica* transcription was down-regulated in the postexponential and stationary phases of planktonic growth. Our results were in concordance with the results obtained by Beenken et al. [5], who did not observe an increase in *icaD* expression at later stages of biofilm formation and found that this gene was significantly upregulated in biofilm when comparing to the stationary phases of planktonic growth. Vandecasteele et al. [21] who analyzed expression of *icaA* and *icaC*, both in vitro and in vivo, also demonstrated that these genes were induced upon initial exposure to foreign bodies and expression peaked shortly after the inoculation of bacteria, after which the expression level slowly decreased over time.

Similarly as reported by Beloin et al. [22], our results also showed that the nature of the strains plays roles in the regulation of biofilm expression. In the case of weakly adhering strain the expression level of *icaA* gene in biofilm grown slowly and peaked at 12 h, while in strongly adhering strain, the highest expression level was found at 3 h. The expression level of *icaD* gene in weakly adhering strain was the same during 12 h and decreased after 24 h, whereas the highest expression level in strongly adhering strain was detected at 3 and 6 h. The differences in expression levels of genes encoding binding factors between strains were also observed. In the case of strongly adhering strain, the expression levels of these genes in biofilm were significantly higher than in weakly adhering strain and the peak expression for the *eno*, *ebps* and *fib* was observed mainly during first 6 h of growth. Atshan et al. [17] who investigated the expression of genes involved in biofilm formation, also observed the differences in expression levels of genes between four MRSA isolates.

## 4. Materials and Methods

### 4.1. Bacterial Strains and Growth Conditions

Three *S. aureus* strains were used for all experiments in this work. The strains from clinical materials from humans, such as swab from wound, respiratory tract, and anus were isolated in 2017. The strains were obtained from hospital in Siedlce (Poland). The presence of the *mecA* gene responsible for resistance against β-lactam antibiotics of these strains was identified by PCR [23].

### 4.2. Biofilm Formation Assay

The strains were grown on Tryptic-Soy Agar (TSA; BBL, Becton Dickinson, Sparks, Md., Franklin Lakes, NJ, USA) with 0.5% glucose at 37 °C for 18 h. After that, 200 µL of bacterial cells suspension of each strains (about 10^8^ CFU/mL) in Tryptic-Soy Broth (TSB) with 0.5% glucose were inoculated in eight replicates to wells of a tissue culture polystyrene 96-well plate (Nunclon, Roskilde, Denmark). Biofilms were developed for 48 h at 37 °C. Subsequently, the medium was removed and the biofilms were washed twice with 250 µL of sterile phosphate buffered saline (PBS, pH 7.4). Biofilms were fixed with 200 µL of methanol per well for 15 min and stained with 200 µL of 1% crystal violet per well (Sigma-Aldrich, Steinheim, Germany). After that, the plates were rinsed with distilled water and air dried. Crystal violet was solved in 96% ethanol to measure absorbance at 492 nm in microplate reader (Apollo LB913, Berthold Technologies, Bad Wildbad, Germany). Each assay was performed three times and the results were averaged. Values of absorbance <0.2 were considered to be weak producers, 0.2–0.4 were medium producers, and values >0.4 were considered strong producers [24].

### 4.3. DNA Isolation

Genomic DNA was isolated from *S. aureus* strains using the NucleoSpin Microbial DNA (Macherey-Nagel GmbH&Co.KG, Düren, Germany), according to the manufacturer’s protocol. 2.5 µL of the total extracted material from each test sample was used as a template DNA for PCR application.

### 4.4. Primers and PCR Conditions

The primers specific for the *icaA*, *icaD*, *eno*, *ebps,* and *fib* synthesized at DNA-Gdańsk (Gdańsk, Poland), are listed in Table 2. The singleplex PCR for each gene was performed in a 25-µL volume containing 2.5 µL of DNA template, 1× PCR buffer, 0.2 mM each dATP, dCTP, dGTP, and dTTP (Fermentas, Vilnius, Lithuania), the specific primers at 100 nM, and 1 U of RedTag Genomic DNA polymerase (Sigma-Aldrich). The amplification was carried out in the following conditions: initial denaturation (94 °C, 5 min), followed by 35 subsequent cycles consisting of denaturation (94 °C, 1 min), primer annealing (56 °C, 1 min), extension (72 °C, 1 min), and final extension (72 °C, 10 min).

Amplifications were carried out in the Eppendorf Mastercycler nexus gradient (Hamburg, Germany). The PCR products were analysed by electrophoresis in 1.5% agarose gels stained with ethidium bromide. Molecular size markers (Sigma-Aldrich) were also run for product size verification. The gel was electrophoresed in 2× Tris-borate buffer at 70 V for 1.5 h. The PCR amplicons were visualized using UV light (Syngen Imagine, Syngen Biotech, Wrocław, Poland).

### 4.5. Growth Conditions of Strains

*S. aureus* strains were grown in Tryptic-Soy Broth (TSB) with 0.5% glucose at 37 °C for 18 h. Subsequently, the cultures were inoculated into fresh TSB with 0.5% glucose and were grown to an optical density of 0.2 at 492 nm. The cultures were diluted to a concentration of about 10^6^ CFU/mL. For planktonic growth conditions, 0.2 mL of the bacterial cell suspension of each strains was transferred to 30 mL of TSB with 0.5% glucose and incubated at 37 °C. For biofilm growth conditions, 0.5 mL of the bacterial cell suspension of each strains was inoculated in eight replicates to wells of a tissue culture polystyrene 24-well plate (Nunclon) and incubated at 37 °C.

### 4.6. Preparation of the Lysate from Bacterial Cells

Planktonically grown and biofilm-grown bacterial cells were harvested at five different times (3, 6, 8, 12, and 24 h). The suspensions of planktonically grown cells (about 10^9^ CFU/mL) (500 µL) were transferred to 1000 µL of RNAprotect Bacteria Reagent (QIAGEN, Hilden, Germany). The biofilm cells were rinsed with sterile water, scraped with pipette, and suspended in appropriate volume of RNAprotect Bacteria Reagent in order to prepare cell suspensions containing about 5 × 10^8^ CFU/mL. Bacterial cell suspensions were intensively vortexed for 5 s and incubated for 5 min at room temperature. After that, the suspensions were centrifuged for 10 min at 5000× *g*. Cells pellets were suspended in 15 µL proteinase K (Macherey-Nagel GmbH&Co.KG, Düren, Germany), vortexed for 10 s, and incubated at room temperature for 10 min. During incubation the mixture was vortexed for 10 s every 2 min. Subsequently, 700 µL RLT buffer (QIAGEN) was added to each bacterial cell suspension, mixed, and transferred to tubes with sterile glass beads (Macherey-Nagel GmbH&Co.KG, Düren, Germany). The bacterial cells with glass beads were vortexed for 5 min and then centrifuged for 20 s at 16,000× *g*. The volume of supernatants was determined and transferred into new tube. Then, the same volume of 70% ethanol was added. The obtained suspensions were used for RNA isolation.

### 4.7. RNA Extraction and cDNA Synthesis

RNA was extracted from lysates of bacterial cells using the RNeasy Protect Bacteria Kit (QIAGEN), according to the manufacturer’s instructions. Quantification of RNA was conducted using an Epoch microplate spectrophotometer (BioTek Instruments, Inc., Winooski, VT, USA). Additionally, A_260/280_ ratios were calculated to evaluate sample integrity and contamination of proteins. Synthesis of cDNA was performed with the use of High Capacity cDNA Reverse Transcription Kit with RNase Inhibitor (Life Technologies, Carlsbad, CA, USA), following the manufacturer’s protocol.

### 4.8. Gene Quantification

Quantification of five tested genes (*icaA*, *icaD*, *eno*, *ebps*, and *fib*) of *S. aureus* strains was performed using real-time qRT-PCR technique. DNA amplification was conducted with application of StepOne Plus Real-Time PCR System and StepOne Plus Software v2.3 (Applied Biosystems, Foster City, CA, USA). Transcript abundance of all five target genes in *S. aureus* cells was achieved using Custom TaqMan Gene Expression Assays, purchased from Life Technologies. *RpoB* gene, encoding RNA polymerase subunit was used as the internal reference (Table 3). The list of primers and TaqMan fluorescent probes that were used in the study is presented in Table 3. The reaction mixture (20 µL) contained 10 µL of 2× TaqMan Fast Universal PCR Master Mix (Life Technologies), 1 µL of 20× Custom TaqMan Gene Expression assay solution, 8 µL of RNase-free water, and 1 µL of cDNA. The following thermal cycling conditions of qRT-PCR amplification were applied: 95 °C for 20 s (activation of AmpliTaq Gold DNA Polymerase (Life Technologies), with subsequent 40 cycles (95 °C for 1 s, 60 °C for 20 s). In order to evaluate the relative expression of five studied genes of *S. aureus*, the comparative *C*_t_ (ΔΔ*C*_t_) method was used [26]. Planktonic cells of *S. aureus* strain (No. 156; at 6 h of growth period) showing moderate biofilm formation were used as the calibrator sample. The obtained results were displayed as *n*-fold changes ± standard deviation (SD) in the target genes expression, in relation to the calibrator.

### 4.9. Statistical Analysis

All data were expressed as the mean ± SD (*n* = 3). The obtained results were assessed with the use of STATISTICA 12 software (StatSoft, Cracow, Poland). Three-factorial analysis of variance (ANOVA) with consecutive Tukey’s test were employed to evaluate the significance of tested variables (strains, cell forms, growth period) and the interactions on expression level of five target genes (*icaA*, *icaD*, *eno*, *ebps*, *fib*) of MRSA strains.

## 5. Conclusions

Our results confirmed that the expression levels of genes that are involved in the synthesis of binding factors and PIA were significantly higher in biofilm than under planktonic conditions. The *eno*, *ebps* and *fib* genes encoding binding proteins were expressed at the highest level in the first phase of biofilm formation. These results showed that bacterial cells, in order to colonize host tissues or foreign body, initially produce large amounts of factors facilitating interaction with host extracellular ligands. The synthesis of the glucosamine polymer by sessile bacterial cells in the first stage of biofilm formation might contribute to the observed persistence and resistance of cells in biofilm. High expression level of genes encoding binding factors and PIA in first phase of biofilm formation decreased with time, which probably was the result of reduced metabolism due to depletion of nutrients and unfavourable oxygen concentrations.

Application of qRT-PCR to examine the transcript levels of the genes involved in adhesion and biofilm formation by MRSA strains was useful for better understanding of the mechanism of biofilm formation over time.

## Figures and Tables

**Table 1:** Characteristics of *Staphylococcus aureus* strains used in study.

Strain	Source	Biofilm	Results of PCR for:					
			*mecA*	*icaA*	*icaD*	*eno*	*ebps*	*fib*
27,887	Wound	Strong	+	+	+	+	+	+
156	Respiratory tract	Moderate	+	+	+	+	+	+
1037	Anus	Weak	+	+	+	+	+	+

**Table 2:** Oligonucleotide primers used in PCR.

Primers	Sequence (5′→3′)	Amplicon Size (bp)	References
*icaA* (F)	ACACTTGCTGGCGCAGTCAA	188	[25]
*icaA* (R)	TCTGGAACCAACATCCAACA		
*icaD* (F)	ATGGTCAAGCCCAGACAGAG	198	[25]
*icaD* (R)	AGTATTTTCAATGTTTAAAGCAA		
*eno* (F)	ACGTGCAGCAGCTGACT	301	[15]
*eno* (R)	CAACAGCATTCTTCAGTACCTTC		
*ebps* (F)	CATCCAGAACCAATCGAAGAC	180	[15]
*ebps* (R)	CTTAACAGTTACATCATCATGTTTATCTTTG		
*fib* (F)	CTACAACTACAATTGCCGTCAACAG	405	[15]
*fib* (R)	GCTCTTGTAAGACCATTTTCTTCAC		

**Table 3:** Sequences of primers designed for real-time qRT-PCR analyses.

Genes	Accession No. (GenBank)	Sequences of Primers and Probes
*icaA*	SAB2541 (K11936)	F: CAATACTATTTCGGGTGTCTTCACTCT R: CAAGAAACTGCAATATCTTCGGTAATCAT P: 5′-FAM-CCCAGTAGCCAACATC-NFQ-3′
*icaD*	SAB2542 (K21461)	F: TCAAGCCCAGACAGAGGGAATA R: ACACGATATAGCGATAAGTGCTGTTT P: 5′-FAM-CCCAACGCTAAAATC-NFQ-3′
*eno*	AF065394.1	F: AAACTGCCGTAGGTGACGAA R: TGTTTCAACAGCATCTTCAGTACCTT P: 5′-FAM-TTCGCTCCTAAATTTG-NFQ-3′
*ebps*	SAB1343c	F: ACATTCAAATGACGCTCAAAACAAAAGT R: CTTATCTTGAGACGCTTTATCCTCAGT P: 5′-FAM-CAAGGCGAATAACTCG-NFQ-3′
*fib*	SAB1021 (K14200)	F: GAATATGGTGCACGTCCACAATT R: AAGATTTTGAGCTTGAATCAATTTTTGTTCTTTTT P: 5′-FAM-TCGCTGCTGGTTTATT-NFQ-3′
*rpoB* (reference)	KY086792.1	F: CAGCTGACGAAGAAGATAGCTATGT R: ACTTCATCATCCATGAAACGACCAT P: 5′-TAGCACAAGCAAACTC-NFQ-3′

## Supplementary Materials

Supplementary materials can be found at http://www.mdpi.com/1422-0067/19/11/3487/s1.
