# Supplementary material for: Expression of the Biofilm-Associated Genes in Methicillin-Resistant Staphylococcus aureus in Biofilm and Planktonic Conditions
Source: Int J Mol Sci. 2018 Nov 6;19(11):3487. doi: 10.3390/ijms19113487 (PMC6274806; doi:10.3390/ijms19113487)
Supplement: Supplementary file 1 [file ijms-19-03487-s001.pdf]

Table S1. Factorial ANOVA analysis of the studied variables (strains, cell forms, growth period) and interactions on expression of five tested genes (*icaA*, *icaD*, *eno*, *ebps*, *fib*) of MRSA strains.

**Parameters**	***icaA***	***icaD***	***eno***	***epbS***	***fib***
Strains (S)	$F_{1, 40}$ = 4,112 (***)	$F_{1, 40}$ = 2,384 (***)	$F_{1, 40}$ = 496 (**)	$F_{1, 40}$ = 1,054 (***)	$F_{1, 40}$ = 815 (**)
Cell forms (C)	$F_{1, 40}$ = 1,675 (***)	$F_{1, 40}$ = 2,157 (***)	$F_{1, 40}$ = 350 (**)	$F_{1, 40}$ = 941 (***)	$F_{1, 40}$ = 1,560 (***)
Growth time (G)	$F_{4, 40}$ = 882 (**)	$F_{4, 40}$ = 1,354 (***)	$F_{4, 40}$ = 642 (**)	$F_{4, 40}$ = 518 (**)	$F_{4, 40}$ = 1,114 (***)
S × C	$F_{1, 40}$ = 409 (**)	$F_{1, 40}$ = 683 (**)	$F_{1, 40}$ = 58 (*)	$F_{1, 40}$ = 296 (**)	$F_{1, 40}$ = 205 (**)
C × G	$F_{4, 40}$ = 143 (*)	$F_{4, 40}$ = 532 (**)	$F_{4, 40}$ = 36 (*)	$F_{4, 40}$ = 148 (**)	$F_{4, 40}$ = 529 (**)
S × G	$F_{4, 40}$ = 206 (**)	$F_{4, 40}$ = 118 (*)	$F_{4, 40}$ = 69 (*)	$F_{4, 40}$ = 99 (*)	$F_{4, 40}$ = 162 (**)
S × C × G	$F_{4, 40}$ = 85 (*)	$F_{4, 40}$ = 60 (*)	$F_{4, 40}$ = 15 (*)	$F_{4, 40}$ = 27 (*)	$F_{4, 40}$ = 53 (*)

* $p < 0.05$; ** $p < 0.01$; *** $p < 0.001$; ns—non-significant. Variability source: (i) strains—weak and strong biofilm formation; (ii) cell forms: PL (planktonic) and BIO (biofilm); (iii) growth period—3, 6, 8, 12 and 24 h.
